# Associations between sleep bruxism and primary headaches: a descriptive study

**DOI:** 10.22514/jofph.2024.038

**Published:** 2024-12-12

**Authors:** Jéssica Conti Réus, Joyce Duarte, Patrícia Pauletto, Helena Polmann, Luiz Paulo de Queiroz, Israel Silva Maia, Graziela De Luca Canto

**Affiliations:** ^1^Brazilian Centre for Evidence Based Research, 88040-900 Florianópolis, SC, Brazil; ^2^Federal University of Santa Catarina, 88040-900 Florianópolis, SC, Brazil; ^3^UniCuritiba University Center, 80220-181 Curitiba, PR, Brazil; ^4^University of the Americas (UDLA), 170517 Quito, Ecuador; ^5^Baia Sul Research Institute, 88020-210 Florianópolis, SC, Brazil

**Keywords:** Sleep bruxism, Polysomnography, Headache, Migraine, Tension-type headache

## Abstract

To evaluate the association between definitive sleep bruxism and primary headaches and to analyze other variables that may also be associated with definitive sleep bruxism. A descriptive study was carried out with a sample of adults with a medical indication for polysomnography in Florianópolis, Brazil. Data were collected in three phases: questionnaires, physical examinations and polysomnography. Pearson’s chi-square test and unadjusted and adjusted binary regressions were carried out using the Statistical Package for the Social Sciences computer program. The significance level was 5%, and the confidence interval (CI) was 95%. The test power was calculated by the G*Power computer program. The sample consisted of 23 men and 19 women, with an average age of 45.6 ± 15 years. Approximately 76% of the participants had sleep bruxism, and 57% had primary headache. The odds ratio between definitive sleep bruxism and primary headaches was 0.86 (95% CI 0.20 to 3.64; *p* = 0.71), demonstrating no association between these variables. Among the other variables analyzed, only alcohol consumption was associated with bruxism, with an odds ratio of 5.96 (95% CI 1.26 to 28.28; *p* = 0.03). According to binary regression, no variable was a confounding factor for definitive sleep bruxism. The power of the test was 0.028. There was no association between definitive sleep bruxism and primary headaches. Alcohol consumption increases the patient’s chance of having sleep bruxism by almost six times. Knowledge about the association of sleep bruxism with other variables can help dentists detect it and explain the condition to patients.

## 1. Introduction

According to the International Classification of Sleep Disorders (ICSD) [1], sleep bruxism is a movement disorder characterized by grinding and/or clenching of the teeth. Additionally, sleep bruxism can be defined as the rhythmic (phasic) or nonrhythmic (tonic) activity of masticatory muscles [2]. Currently, sleep bruxism is not considered a dysfunction in healthy individuals but rather a behavior with potential clinical consequences [2].

Sleep bruxism can be categorized according to the diagnostic method as possible, probable or definite [2]. Possible sleep bruxism can be detected by self-reports, probable by clinical exams (intra- or extraoral), and definitive sleep bruxism can be detected by diagnostic devices, such as electromyography (EMG) or polysomnography (PSG). The prevalence of possible sleep bruxism is approximately 12.8% (±3.1%) in adults [3]. However, considering only PSG as the diagnostic criterion, the prevalence of possible sleep bruxism was 7.4% in a Brazilian population sample [4].

The etiopathogenesis of bruxism is multivariate and originates in the central nervous system [5]. For this reason, most of the papers published on the subject have focused on associations with systemic conditions or habits linked to the central nervous system, such as hormone production and receptor [6, 7], genetics [8], screen-time [9], caffeine intake [10], alcohol consumption [10, 11], stress [12], sleep apnea [13] and headaches [14, 15].

Headaches can be classified as primary or secondary [16]. Primary headaches comprise a broad spectrum of painful conditions, the most common of which are migraine and tension-type headache (TTH) [17, 18]. The main differences between migraine and TTH are related to the location, duration, quality and intensity of the pain [16]. The prevalence of migraines is approximately 14%, and that of TTH is approximately 26% [19]. The diagnosis of headaches is carried out through dental and medical history, questionnaires and physical examination, which a neurologist must interpret [16].

Because there are many primary articles that study the association between sleep bruxism and primary headaches, a systematic review published in 2021 [14] only included studies that had diagnosed primary headaches using the International Classification of Headache Disorders (ICHD) [16] (regardless of edition). Even so, studies remained heterogeneous and this heterogeneity comes from faulty diagnostic methods for sleep bruxism, resulting in a gap in the literature, highlighting the necessity of current studies on this with better and reliable methodology. Hereby, the primary objective of this study was to verify the association between definite sleep bruxism and primary headaches. The secondary objective was to evaluate the interference of sleep apnea, coffee and alcohol consumption, and cigarette use in definite sleep bruxism detection. The null hypothesis (H0) is that there is no association and the alternative hypothesis (H1) is that there is an association between sleep bruxism and primary headaches.

## 2. Methods

### 2.1 Study design, setting and participants

This descriptive study was reported following the “Strengthening the Reporting of Observational Studies in Epidemiology” (STROBE) [20].

Individuals referred to a night-time PSG upon prior medical request at Baia Sul Hospital, a private hospital located in Florianópolis, SC, Brazil, were invited to participate. The sleep laboratory of Baia Sul Hospital performed PSG on alternate days, including weekends. The same PSG technician was responsible for overseeing the procedure, which was consistently performed in the same room. When we started our study, our plan was to collect data from a whole year; however, we stopped collecting data in October 2019 because the technician resigned. We were waiting for replacement when the Corona virus disease pandemic emerged and the sleep laboratory was closed. Consequently, we collected data only from a nine-month period. Given the nonprobabilistic nature of the sample, a sample size calculation was not performed [21, 22].

### 2.2 Inclusion criteria

Adult patients (≥18 years) scheduled for PSG without self-reported neurological impairment.

### 2.3 Exclusion criteria

Patients with incomplete physical or PSG exams.

### 2.4 Data sources and measurements

The data collection was divided into three stages: (1) application of electronic questionnaires, (2) physical examination and (3) PSG. One of the three researchers (JCR, HP and PP) individually conducted all questionnaires and physical exams in the hospital bedroom where the patients were admitted, just prior to the PSG exam.

#### 2.4.1 Electronic questionnaires

An electronic questionnaire created on Google Docs (**Supplementary material 1**) was self-completed by the patient on a tablet (iPad Air 2, Apple®, Cupertino, CA, USA).

Data regarding sex, age, height, weight, schooling and primary headaches were collected in this first stage; in addition, the following dichotomous variables (yes/no) were collected: consumption of coffee, consumption of alcoholic beverages and cigarette smoking habit. Furthermore, the ordinal variables of quantity of coffee and smoking habit (1 to 3 cups/cigarettes per day; 4 to 6 cups/cigarettes per day; more than 7 cups/cigarettes per day), alcohol consumption (1 to 3 times per week; 4 to 6 times per week; more than 7 times per week) and how long the patient has had this habit (1 to 3 years; 4 to 6 years; and more than 7 years) were also recorded.

Primary headaches were diagnosed based on answers from the questionnaires. The questions were developed by the authors (see **Supplementary material 1**) based on the characteristics of each primary headache mentioned in the third edition of the ICHD [16]. The final diagnosis was performed by a neurologist (LPQ), who was blinded to the study, analyzed the patients’ answers, and categorized the diagnoses as “migraine” or “tension-type headache”. Additionally, both types of headaches were subclassified as “probable” or “definitive”. For the diagnosis to be considered “definitive”, all items with the characteristics of each primary headache had to be selected by the patient. Headache was considered “probable” when the patient did not fulfill all the characteristics for a given definitive classification according to the ICHD [16]. The features of both types of headaches are as follows:

Characteristics of migraine: duration between four and 72 hours, unilateral location, pulsating quality, moderate or severe intensity, exacerbation by daily life activities, association with nausea and/or vomiting and/or photophobia and phonophobia.

Characteristics of TTH: duration between 30 minutes and 7 days, typically bilateral, pressing, or dull quality, mild to moderate intensity and no worsening of pain during daily life activities; TTH is not associated with nausea or vomiting, although photophobia or phonophobia may be present.

#### 2.4.2 Physical exam

A calibrated digital scale (Digital Glass G-Tech, ACCUMED) was used to measure the patient’s weight in kilograms. A tape measure was used to measure the patient’s height. For measurement, the patient stood with his or her arms extended along the body and spine straight, his or her eyes at a fixed point looking straight ahead, and heels and knees spread evenly apart with their buttocks against the wall. Weight and height were measured and recorded by the researcher responsible for data collection on the day of the PSG exam. The body mass index ratio (BMI) was calculated by dividing weight by height squared using a formula in Microsoft® Excel 16.29.1 (Microsoft Office 2019, Microsoft, Redmond, WA, USA).

#### 2.4.3 Polysomnography

Single overnight sleep exams were conducted in a dark, quiet hospital room using a conventional PSG testing equipment (Alice V; Respironics, Andover, MA, USA), following the recommendations proposed by American Academy of Sleep Medicine (AASM) [23]. The instruments were calibrated for an adequate recognition of the signs. The participants were instructed, before the beginning of the records, to perform voluntary snoring movements, leg movement, opening, closing and moving the eyes, teeth clenching, mandibular laterality, mandibular protrusion, as well as swallowing and coughing movements to record baseline amplitudes. Sleep recordings were registered with surface electrodes at the following locations. Three electroencephalogram (EEG) channels: (F4-M1, C4-M1, O2-M1) and other 3 back up leads (F3-M2, C3-M2, O1-M2; 2). Two electrooculography (EOG) channels: E1-M2 leads (E1 placed 1 cm below and 1 cm lateral to the distal end of the left eye and E2-M2 placed 1 cm below and 1 cm lateral to the distal end of the right eye). Three electrodes for electromyography (EMG): central position 1 cm above the lower edge of the mandible, another 2 cm below the lower edge of the mandible 2 cm to the right of the midline and another in the same position, but to the left of medium line; Leg EMG with 1 electrode on each leg in the anterior tibial region to be recorded in just 1 channel.

To detect definite sleep bruxism, two electrodes were placed in the masseter region, one on each side. The events were estimated through rhythmic masticatory muscle activity (RMMA), which was evaluated through EMG. Phasic or tonic elevations in masseter myographic activity of at least twice the amplitude of the baseline recording were noted. Events separated by 3-second intervals were considered sleep bruxism episodes if they followed one of the following patterns: (1) tonic—at least one masseteric EMG burst greater than 2 seconds; (2) phasic—three or more shots of masseteric EMG lasting between 0.25 and 2 seconds; or (3) mixed. Participants whose bruxism episode index was more than two episodes per hour of sleep were considered to have definite sleep bruxism [1]; those whose index was between two and four were categorized as mild/moderate, and those whose index was more than four were categorized as severe.

The sleep apnea diagnosis was also performed by PSG and exclusively based on apnea-hypopnea index (AHI) values. A sleep apnea event was determined when there was an interruption in airflow ≥90% for a minimum of 10 seconds. Mild sleep apnea was defined as an AHI between 5 and 15 episodes per hour of sleep, moderate sleep apnea was defined as an AHI between 15 episodes and fewer than 30 events per hour, and severe sleep apnea was defined as an AHI greater than 30 events per hour [23].

Sample selection bias was present because all patients who underwent PSG had suspected sleep-related problems. However, data collection bias was controlled since each participant had a medical record number that was completed in the electronic form and in the PSG exam. The PSG exams were also performed by a sleep specialist pulmonologist (IMS), and the analyses were performed by a single trained evaluator (JD) who was blinded to the questionnaires and physical examination results.

## 3. Statistical methods

The statistical analysis was performed using the software Statistical Package for Social Sciences 2021 (IBM Corp, Armonk, NY, USA). Quantitative (age) and qualitative (sex, definite sleep bruxism, primary headaches, apnea and coffee, alcohol and cigarette use) variables are shown as averages (with standard deviation) and frequencies (in percentage form), respectively. Pearson’s chi-square test was used to calculate the odds ratio (OR) for dichotomous variables (*e.g.*, definite sleep bruxism, primary headaches and coffee, alcohol and cigarette use). They were introduced into the computer program with the following categories: “no” (0) and “yes” (1).

To detect potential variables that could interfere with definite sleep bruxism diagnosis, unadjusted and adjusted binary regression (insert method) was performed. Adjustments were made in the unadjusted model for the independent variables that presented a *p* value < 0.2 in the unadjusted analysis [24, 25]. Primary headaches and sleep apnea were independently included in the adjusted model. The first reason is the main variable studied; the second reason is that patients were referred for PSG examination for suspected breathing or sleep problems. The level of significance was set at 5%. ORs and 95% confidence intervals (CIs) were calculated.

The test power (1 − β err) was calculated through a *post hoc* test in G*Power software version 3.1.9.7 using the proportion of patients with primary headache among the bruxer and no bruxer groups. A one-tailed value with an α err of 0.05 was considered.

## 4. Results

### 4.1 Participants and descriptive data

Among the participants who agreed to participate in the research, 42 patients (60.9%) had a PSG exam completed, and the primary headache was diagnosed (Fig. 1). Twenty-three patients were males (mean age: 40.7 ± 13.7 years), 19 were females (mean age: 51.6 ± 14.6 years) and they were aged 21 to 80 years (mean age: 45.6 ± 15 years). Fifty percent of the participants were obese according to their BMI (≥30). The demographic characteristics of the sample are presented in Table 1.

**Fig. 1.** Flowchart of the study sample number of participants and missing data diagram. PSG: Polysomnography.

**Table 1.** Demographic and studied variables’ characteristics of the sample.

		Male (n = 23)	Female (n = 19)	Total (n = 42)
Age (yr)				
	20–29	4 (17.4%)	1 (5.3%)	5 (11.9%)
	30–39	8 (34.8%)	4 (21.1%)	12 (28.6%)
	40–49	8 (34.8%)	2 (10.4%)	10 (23.8%)
	50–59	0	6 (31.6%)	6 (14.3%)
	60 and over	3 (13.0%)	6 (31.6%)	9 (21.4%)
Schooling				
	Under graduation	7 (30.4%)	10 (52.3%)	17 (40.5%)
	Graduation	11 (47.8%)	6 (31.6%)	17 (40.5%)
	Post-graduation	5 (21.8%)	3 (16.1%)	8 (19.0%)
Definite Bruxism				
	No	5 (21.8%)	5 (26.3%)	10 (23.8%)
	Yes	18 (78.2%)	14 (73.7%)	32 (76.2%)
Headache				
	No headache	12 (52.2%)	6 (31.6%)	18 (42.9%)
	Definitive migraine	0	6 (31.6%)	6 (14.3%)
	Probable migraine	1 (4.3%)	3 (15.8%)	4 (9.5%)
	Definitive TTH	6 (26.1%)	3 (15.8%)	9 (21.4%)
	Probable TTH	4 (17.4%)	1 (5.3%)	5 (11.9%)
Apnea				
	No	5 (21.8%)	3 (15.8%)	8 (19.0%)
	Yes	18 (78.2%)	16 (84.2%)	34 (81.0%)
Alcohol				
Yes (beer/wine)		19 (13/6) (82.6%)	7 (6/1) (36.8%)	26 (61.9%)
Duration	<3 yr	0	1 (14.3%)	1 (3.9%)
	3 to 6 yr	2 (10.5%)	1 (14.3%)	3 (11.5%)
	≥7 yr	17 (89.5%)	5 (71.4%)	22 (84.6%)
Frequency	1 to 3 times/wk	16 (84.2%)	6 (85.7%)	22 (84.6%)
	4 to 6 times/wk	2 (10.5%)	1 (14.3%)	3 (11.5%)
	≥7 d/wk	1 (5.3%)	0	1 (3.9%)
Smoking				
Yes		2 (8.7%)	2 (10.5%)	4 (9.5%)
Duration	<3 yr	1 (50.0%)	0	1 (25.0%)
	4 to 6 yr	1 (50.0%)	0	1 (25.0%)
	≥7 yr	0	2 (100.0%)	2 (50.0%)
Quantity	1 to 3 cigarette/d	1 (50.0%)	0	1 (25.0%)
	≥7 cigarettes/d	1 (50.0%)	2 (100.0%)	3 (75.0%)
Coffee				
Yes (without milk/with milk)		22 (14/8) (95.7%)	17 (5/12) (89.5%)	39 (92.9%)
Duration	<3 yr	1 (4.5%)	0	1 (2.6%)
	4 to 6 yr	0	1 (5.9%)	1 (2.6%)
	≥7 yr	21 (95.5%)	16 (94.1%)	37 (94.8%)
Quantity	1 to 3 cups/d	17 (77.3%)	13 (76.5%)	30 (76.9%)
	4 to 6 cups/d	4 (18.2%)	4 (23.5%)	8 (20.5%)
	≥7 cups/d	1 (4.5%)	0	1 (2.6%)

*TTH: tension-type headache; yr: years; wk: week; d: day.*

### 4.2 Outcomes

According to the PSG results, 32 patients (56.3% men) had definite sleep bruxism (76.2%) and among them, 14 patients (43.8%) had severe sleep bruxism.

Thirty-four patients were diagnosed with sleep apnea (80.9%), and among them, 23.5%, 53% and 23.5% had mild, moderate and severe sleep apnea, respectively.

Twenty-four patients (57.1%) were classified as having primary headaches. Six (25%) patients were diagnosed with definitive migraine, nine (37.5%) with definitive TTH, four (16.7%) with probable migraines and five (20.8%) with probable TTH. Men were more affected by TTH than women, with a proportion of 4:1 in probable TTH patients and 2:1 in definitive TTH patients. All six definitive migraine cases were found in women. Among those with probable migraine, women were more affected than men, at a proportion of 3:1.

In total, 19 of 23 men (82.6%) and seven of 19 women (36.8%) reported a habit of consuming alcohol (wine or beer). Among them (n = 26), 84.6% drank one to three times per week and the same percentage (but not necessarily the same patients) drank for more than seven years. Only four patients smoked cigarettes, with an equal sex distribution. Thirty-nine (56.4% of whom were men) drank coffee.

### 4.3 Main results

#### 4.3.1 Definite sleep bruxism × primary headaches

Eighteen bruxer patients (56.3%) were diagnosed with primary headache, four with migraine, one with probable migraine, eight with TTH and four with probable TTH. Among the ten patients without bruxism, 60% had primary headaches.

The OR between definite sleep bruxism and primary headaches was 0.86 (95% CI 0.20 to 3.64), showing no association between these variables. Additionally, when calculating the OR for each type of primary headache separately, there was no association. More details can be found in Table 2.

**Table 2.** Association between definite sleep bruxism and primary headaches.

	Definite Sleep Bruxism		Odds Ratio (95% CI)
	Presence	Absence	
No headache	14	4	Reference
Migraine	4	2	0.57 (0.08 to 4.35)
Both migraines	6	4	0.36 (0.06 to 2.00)
TTH	8	1	2.29 (0.22 to 24.14)
Both TTH	12	2	1.71 (0.27 to 11.06)

*TTH: tension-type headache; CI: confidence interval.*

#### 4.3.2 Definite sleep bruxism × coffee, alcohol and cigarette habits

The prevalence of bruxism among individuals who consumed alcohol was 71.9%. Conversely, of the individuals without bruxism, only 30% (n = 3) consumed alcohol. All (100%) individuals without bruxism consumed coffee (n = 10), while 90.6% (n = 29) of the individuals with sleep bruxism consumed coffee. Only four sleep bruxism patients smoked, and none of the individuals without bruxism smoked.

The analysis was performed with all secondary variables (sleep apnea and coffee, alcohol and cigarette habits). An association occurred only between definitive sleep bruxism and alcohol consumption (OR 5.96, 95% CI 1.26 to 28.28). The ORs between sleep bruxism and coffee intake and between sleep bruxism and cigarette usage could not be calculated because in the group without sleep bruxism, no patients smoked and all of them drank coffee. All OR analyses are shown in Table 3.

**Table 3.** Unadjusted and adjusted logistic binary regression for association between definite sleep bruxism and independent variables.

Variables	Unadjusted Odds Ratio (95% CI)		Adjusted Odds Ratio (95% CI)	
	With definite sleep bruxism	*p* value	With definite sleep bruxism	*p* value
Age	1.02 (0.97 to 1.07)	0.51		
Sex^a^	0.46 (0.11 to 1.94)	0.29		
Schooling*	2.21 (0.88 to 5.52)	0.09	1.69 (0.61 to 4.68)	0.31
Primary headache^b^	0.86 (0.20 to 3.64)	0.71	1.31 (0.26 to 6.64)	0.74
Apnea^b^	1.08 (0.18 to 6.46)	0.93	0.92 (0.13 to 6.50)	0.93
Alcohol*^, b^	5.96 (1.26 to 28.28)	0.03	4.73 (0.88 to 25.37)	0.07

*Note: Variables “primary headache” and “apnea” were kept in adjusted model; the first is the main variable of this study and the second is due to those patients were referred to the PSG exam for suspected breathing or sleep problems.* *Variables with a significance level p ≤ 0.2 were kept in the model to control for confusion. *^a^Reference: male.* *^b^Reference: no.* *CI: confidence interval.*

#### 4.4 Other analyses

##### 4.4.1 Regression

Beyond primary headaches and sleep apnea, after calculating the OR and *p* value of all variables in the unadjusted binary regression, only schooling and alcohol consumption were considered for the adjusted regression. Nevertheless, the adjusted regression model showed that no variable influenced the presence of sleep bruxism. Cigarette smoking and coffee ingestion were not included in this analysis because the OR could not be calculated. More details are provided in Table 3.

##### 4.4.2 Sensitivity analysis

Sensitivity analysis was performed for the OR between definitive sleep bruxism and sleep apnea since it is considered a variable with potential bias on the result, and most patients underwent PSG to confirm the diagnosis, representing almost 81% of the sample population. The OR was 1.08 (95% CI 0.18 to 6.46), showing no influence on the primary outcome.

##### 4.4.3 Test power

The test power was low, at 0.028.

## 5. Discussion

The main objective of this study was to assess the association between definite sleep bruxism and primary headaches (migraine and TTH). The hypothesis of this study was that the primary headaches and bruxism symptoms originated from the central nervous system [26, 27]. In other words, the same factor that triggers sleep bruxism can trigger headaches as a result; the opposite is also true. However, an association between these two variables was not found.

An article that diagnosed sleep bruxism through PSG concluded that it has an impact on the severity of headaches [15]. In contrast, a recent systematic review [14] concluded that sleep bruxism was not associated with TTH [28, 29] and that this relationship between sleep bruxism and migraines was questionable, as one study showed an association [30] and another did not [28]. In contrast, some studies have concluded that awake bruxism is associated with primary headaches [29, 31, 32] since persistent daytime clenching increases tension in the masticatory muscles, resulting in sensitization of peripheral muscle nociceptors and consequently the occurrence of a headache [33, 34, 35].

A point to highlight in this research is the use of a type I PSG exam for bruxism diagnosis. This subdivision is the most complete (when compared to types II, III and IV), demanding a sleep laboratory or hospital with electrode placement in some parts of the patient’s body and capable of detecting various sleep disorders [36]. In 2018, a systematic review [37] comparing PSG (type I) and portable sleep monitoring (type IV) for diagnosing obstructive sleep apnea (OSA) was published. The authors concluded that the diagnostic accuracy of type IV disease varied depending on the number of channels, setting and disease severity, and it could only help to expand access to early OSA identification and timely management. Although PSG is considered the reference standard for diagnosing sleep bruxism, it is not widely used. The main reasons are the high cost and acceptable diagnostic accuracy of other diagnostic methods that are more accessible to patients [38, 39].

This paper focused on TTH and migraine because of their greater prevalence than other types of headaches and are considered two of the most prevalent neurological disorders worldwide [40]. The diagnosis was made according to the third edition of the ICHD. The prevalence of both types of tension headaches in the overall sample (33.3%) was greater than that of migraines (23.8%), which is consistent with the findings of previous studies [41, 42, 43, 44]. Although more than half of the individuals with bruxism had some type of primary headache (56.3%), mainly tension-type headache, 60% of the individuals without bruxism also had primary headache, justifying the absence of any association.

Even though it was not a primary outcome, obstructive sleep apnea was considered important for this analysis since most patients with suspected sleep breathing problems went to the hospital. Although the association between sleep bruxism and sleep apnea has been extensively studied in the current literature, it is still controversial. Some studies showed an association [45, 46], while others found no association or did not have enough scientific evidence to support the association [13, 47, 48, 49]. The main reason is the heterogeneity in diagnosing sleep apnea and sleep bruxism and the metrics used to detect them. In the present study, this association was not found.

Regarding other analyzed variables, despite the high variation in the confidence interval (resulting in real uncertainty in the OR value), there was only an association with alcohol consumption, agreeing with a previous primary study [49, 50] and systematic review [10]. In individuals with preexisting breathing-related disorders, alcohol consumption worsens respiratory events and lowers the oxygen saturation level [51, 52]. Furthermore, alcohol, which is consumed mainly at night, can affect the regular distribution of sleep stages, causing acute increases in the local concentrations of serotonin, opioids and dopamine in the brain [53, 54]. The presence of these hormones increases the duration of masseter EMG by more than five seconds, suggesting an increase in sleep bruxism events [55]. Despite revealing an association between definitive sleep bruxism and alcohol consumption, when alcohol consumption was controlled for other variables, this association was lost. This change in the odds ratio and the *p* value of the alcohol variable can also be noted in a recent publication [56]. In addition, the same systematic review [10] showed an association of sleep bruxism with coffee consumption and with cigarette smoking, agreeing with two other recent articles regarding these two variables [11, 57]. In this study, due to the small sample size, the ORs of those variables could not be calculated since very few people who smoked and who did not drink coffee were included in the sample. Only four patients (9.5%) reported that they smoked. This number is consistent with research carried out in 2021 on the Brazilian population, where the prevalence of smoking in adults was 9.1% [58]. Similarly, according to the International Coffee Organization [59], Brazil consumed more coffee in 2021 when compared with other countries in the world, totaling at 1,344,000 kilos during 2021.

The present study had several limitations. Sample selection bias was not controlled since the participants invited to participate in the research had already been referred by their physicians to undergo PSG for suspected sleep-related problems. The subjects spent only one night in the hospital for PSG exams, as it was convenience-based and lacked funding for a second night. The literature recommends at least two nights, since the quality of sleep is better on the second night, potentially impacting the RMMA frequency [60, 61]. Although some articles report no association between sleep bruxism and temporomandibular disorders (TMD) or orofacial pain [62, 63, 64], the lack of diagnosis of TMD in the sample resulted in the non-evaluation of muscle pain, whether myalgia or myofascial pain, which can be considered a factor of confusion for the study, since painful TMDs are associated with headaches [65]. Other confounding factors to be mentioned are the presence of anxiety, stress, depression and the administration of controlled medications [66]. They all modulate the central nervous system and can interfere with the diagnosis of both sleep bruxism and primary headaches. Moreover, the small number of patients—a phenomenon associated with the Corona virus disease pandemic—resulted in low test power (when compared to the ideal minimum of 0.8), and coffee and cigarette variables could not be analyzed, as almost all individuals in the samples drank coffee, and only a few of them smoked. Furthermore, the results from convenience samples will probably not be generalizable, which prevents statistical inference for a larger population [21, 22] (therefore impairing the external validity). However, despite the interruption of the study, we have decided that the data should be published, as there are few studies with a robust sample of patients who underwent PSG to analyze sleep bruxism. It is worth highlighting that more similar studies with larger samples must be conducted to extrapolate the findings to the general population.

## 6. Conclusions

Based on a small and convenience sample of individuals with suspected sleep-related problems, there was no association between definitive sleep bruxism and primary headaches (either migraine or TTH). Despite the high variation in the confidence interval, alcohol consumption increased the patient’s chance of having sleep bruxism by almost six times. The analyses also revealed no associations between sleep bruxism and sex, age or apnea.

## Supplementary Material


