# Supplementary material for: Associations between sleep bruxism and primary headaches: a descriptive study
Source: J Oral Facial Pain Headache. 2024 Dec 12;38(4):52–60. doi: 10.22514/jofph.2024.038 (PMC11810669; doi:10.22514/jofph.2024.038)
Supplement: Supplementary file 1 [file Supplementary-material.docx]

Supplementary material

Supplementary material 1. Electronic questionnaires.

**Formulário Eletrônico**

**Dados Do Participante**

Gostaríamos de agradecer ao sr. (a) pela participação em nossa pesquisa. Sua identidade não será revelada e os dados serão mantidos em sigilo.

*Obrigatório

1. Qual seu número de identificação? (número) *

_______________________________

2. Qual a sua idade? (anos) *

_______________________________

3. Qual a sua altura? (centímetros) *

­­_______________________________

4. Qual o seu peso? (quilogramas) *

_______________________________

5. Qual seu estado civil? *

☐ Solteiro(a)

☐ Casado(a)

☐ Separado(a)

☐ Viúvo(a)

☐ União estável

6. Qual o seu grau de escolaridade? *

☐ Ensino fundamental completo

☐ Ensino médio completo

☐ Ensino superior completo

☐ Pós-graduação

7. Qual a sua faixa de renda salarial? *

☐ De 1 a 3 salários mínimos (R$954,00–R$2862,00)

☐ De 3 a 6 salários mínimos (R$2863,00–R$5724,00)

☐ De 6 a 9 salários mínimos (R$5725,00–R$8586,00)

☐ Mais de 10 salários mínimos (Mais de R$9540,00)

**Consumo de bebidas alcoólicas**

8. Você possui o hábito de ingerir bebidas alcoólicas? *

☐ Não Vai para a questão 12.

☐ Sim

9. Qual tipo de bebida alcoólica você costuma ingerir? *

☐ Cerveja

☐ Destilados (cachaça, rum, tequila, uísque, vodka) Vinho

10. Há quanto tempo você tem o hábito de ingerir bebidas alcoólicas? *

☐ Menos de 1 ano

☐ De 1 a 3 anos

☐ De 3 a 6 anos

☐ De 6 a 9 anos

☐ Mais de 10 anos

11. Qual a frequência semanal de consumo de bebidas alcoólicas? *

☐ 1 a 3 vezes por semana

☐ 3 a 6 vezes por semana

☐ Mais de 7 vezes por semana

**Hábito de fumar**

12. Você possui o hábito de fumar? *

☐ Não Vai para a questão 16.

☐ Sim

13. Qual tipo de fumo? *

☐ Cachimbo

☐ Cigarro de palha

☐ Cigarro comum

☐ Cigarro eletrônico

☐ Charuto

☐ Narguilé

14. Há quanto tempo você tem o hábito de fumar? *

☐ Menos de 1 ano

☐ De 1 a 3 anos

☐ De 3 a 6 anos

☐ De 6 a 9 anos

☐ Mais de 10 anos

15. Quantas vezes por dia você costuma fumar? *

☐ 1 a 3 vezes por dia

☐ 3 a 6 vezes por dia ☐ Mais de 7 vezes por dia

**Hábito de tomar café**

16. Você possui o hábito de tomar café? *

☐ Não Vai para a questão 20.

☐ Sim

17. Como você costuma tomar café? *

☐ Café com leite

☐ Café puro

18. Há quanto tempo você tem o hábito de tomar café? *

☐ Menos de 1 ano

☐ De 1 a 3 anos

☐ De 3 a 6 anos

☐De 6 a 9 anos

☐ Mais de 10 anos

19. Quantas vezes por dia você costuma tomar café? *

☐ 1 a 3 vezes por dia

☐ 3 a 6 vezes por dia

☐ Mais de 7 vezes por dia

**Questionário Cefaleias Primárias**

36. 1. Você tem ou já teve dor de cabeça? *

☐ Não

☐ Sim

37. 2. Quantas vezes, por mês, você tem dor de cabeça (crises)? *

☐ Até 2 vezes

☐ De 3 a 5 vezes

☐ Mais de 10 vezes

☐ Outro ________________

38. 3. Assinale os sinais e sintomas que acompanham a sua dor de cabeça: (pode mais de uma opção)

☐ Unilateral (um lado da cabeça)

☐ Bilateral (dois lados da cabeça)

☐ Dor pulsátil

☐ Dor de pressão/apertamento

☐ Dor ligeira a moderada

☐ Dor moderada a grave

☐ Dor grave a muito grave

☐ Fotofobia (aversão a luz)

☐ Fonofobia (aversão a sons)

☐ Dor nos olhos (superfície ou profundo)

☐ Dor no músculo temporal (nas “têmporas”)

☐ Sensação de inquietação

☐ Olhos vermelhos e lacrimejo

☐ Congestão nasal

☐ Sensação de ouvido cheio

☐ Olhos inchados

☐ Agravamento da dor por atividades de rotina

☐ Não há agravamento da dor por atividades de rotina

☐ Náusea e/ou vômito

☐ Queda da pálpebra superior

Supplementary material 2. Free and informed consent form.

UNIVERSIDADE FEDERAL DE SANTA CATARINA

CENTRO DE CIÊNCIAS DA SAÚDE

PROGRAMA DE PÓS-GRADUAÇÃO EM ODONTOLOGIA

**TERMO DE CONSENTIMENTO LIVRE E ESCLARECIDO**

Eu, Joyce Duarte, doutoranda do Programa de Pós-Graduação em Odontologia, do Centro Ciências da Saúde da Universidade Federal de Santa Catarina (UFSC), sob orientação da Prof.ª Dr.ª Graziela De Luca Canto, convido você a participar da pesquisa intitulada “Associação entre bruxismo do sono e síndrome da apneia e hipopneia obstrutiva do sono em adultos”.

O objetivo deste documento é dar a você informações suficientes sobre a pesquisa a qual você está sendo convidado a participar.

# Objetivo Do Estudo

Este estudo tem por objetivo avaliar se existe associação entre o bruxismo do sono, caracterizado por rangimento ou apertamento dos dentes durante o sono e a síndrome da apneia e hipopneia obstrutiva, um problema respiratório onde há bloqueio total ou parcial da respiração durante o sono, através de dados obtidos por exame físico, questionários e exames de polissonografia.

# Procedimentos

● Em um primeiro momento, serão aplicados questionários específicos para identificar sinais e sintomas de bruxismo do sono e síndrome da apneia e hipopneia obstrutiva do sono.

● Será realizado um exame físico da sua boca e músculos da mastigação e posteriormente uma raspagem da mucosa oral, para avaliar as possíveis causas genéticas do Bruxismo do Sono. O exame físico e raspagem da mucosa oral terão a duração entre 5 e 10 minutos. O exame físico é como qualquer exame odontológico de rotina, e não terá nenhuma consequência. Se não se sentir à vontade para a realização do exame, este será interrompido imediatamente. Os usos das células da mucosa bucal serão unicamente para essa pesquisa e saliento que logo após essa análise, elas serão descartadas de forma apropriada.

● Serão coletados também dados demográficos básicos como sexo, idade, estado civil e ocupação. Poderão também ser requisitadas outras informações, tais como peso, altura ou informações relevantes para o tema de pesquisa.

● Se constatadas necessidades de tratamento odontológico durante a avaliação clínica, você será orientado a buscar tratamento nas clínicas odontológicas da Universidade Federal de Santa Catarina.

● Toda a pesquisa envolve riscos, por isto se você não se sentir confortável para realizá-la, poderá desistir a qualquer momento. Nesta pesquisa os riscos são: desconforto ao realizar o exame clínico bucal, cansaço e aborrecimento em responder os questionários, quebra de sigilo, ainda que involuntário e não intencional. Para minimizar o risco de quebra de sigilo, seus dados terão um número para identificação, e não o seu nome. No entanto, como benefício direto, você terá uma avaliação clínica e levantamento de necessidades odontológicas, assim como os devidos encaminhamentos para o serviço. Como benefício indireto, você poderá contribuir para a elucidação e compreensão da fisiopatologia do bruxismo do sono e da apneia e hipopneia obstrutiva do sono.

● Os procedimentos da pesquisa (exame físico e aplicação de questionários), bem como os materiais utilizados, serão custeados pela Instituição proponente da pesquisa (UFSC).

# Participação Voluntária

Sua participação nesse estudo não é obrigatória e não haverá custos nem pagamentos pela mesma. Uma vez que você decidiu participar do estudo, você pode retirar seu consentimento de participação a qualquer momento, sem prejuízos de qualquer natureza. Não haverá reembolso, uma vez que com a participação na pesquisa você não terá custo.

# Justificativa Do Estudo

O estudo servirá para a compreensão dos fatores associados ao bruxismo do sono, ou seja, se problemas respiratórios do sono causam, ou não, o ranger/apertar de dentes durante o sono.

# Permissão Para Revisão De Registros, Confidencialidade E Acesso Aos Registros

Será solicitada permissão para acesso aos registros dos exames de polissonografia realizados pelo hospital. Sua identidade não será revelada e os dados serão analisados e mantidos em sigilo. Os dados obtidos por meio dos questionários, exames físico e exames de polissonografia serão utilizados em publicações futuras e você terá acesso a eles a qualquer momento da pesquisa.

# Contato Com Os Pesquisadores

Se você tiver alguma dúvida em relação ao estudo, você deverá entrar em contato com a pesquisadora do estudo Joyce Duarte, pelo telefone (48) 3721-4952 ou *via* e-mail: joyceduarteortodtm@gmail.com ou da pesquisadora responsável, Graziela De Luca Canto, professora do Departamento de Odontologia, Centro de Ciências da Saúde – UFSC (Campus Trindade), telefone (48) 3721-4952 e e-mail delucacanto@gmail.com. Esta pesquisa atende a Resolução do CNS 466/2012 e conta com a aprovação do CEPSH/UFSC (Rua Desembargador Vitor Lima, nº 222, Trindade, Florianópolis, Prédio Reitoria II, 4º andar, sala 401). Caso você apresente alguma dúvida em relação a questões éticas, o contato com o Comitê de Ética dessa Instituição pode ser realizado por meio do telefone (48) 3721-9206 ou e-mail: cep@reitoria.ufsc.br.

# Declaração Do Pesquisador

A pesquisadora responsável por esta pesquisa, Prof.ª Dr.ª Graziela De Luca Canto, se compromete a seguir a Resolução CNS n. 466/12 em todos os seus itens, entre os quais destacam-se: ressarcimento ou indenização de custos gerados em função da pesquisa, desde que estes sejam comprovados. O suporte, custeio, ressarcimento ou indenização serão de responsabilidade dos pesquisadores deste projeto, seguindo o que rege a resolução CNS n. 466/12.

# Declaração De Consentimento Do Paciente

Eu_________, CP_________, RG_________, residente à _____________________, estou ciente que me é garantido o livre acesso a todas as informações e esclarecimentos adicionais sobre o estudo durante e depois da minha participação. Declaro ter sido informado e estar devidamente esclarecido sobre os objetivos deste estudo. Recebi garantias de total sigilo e de obter novos esclarecimentos sempre que desejar, assim como afirmo também ter recebido uma *via* do presente Termo de Consentimento Livre e Esclarecido. Assim, concordo em participar voluntariamente deste estudo e sei que posso retirar meu consentimento a qualquer momento, sem qualquer prejuízo.

Florianópolis_______________/________________/_____________

_______________________________________________________

Assinatura do participante

_______________________________

Assinatura da pesquisadora: Joyce Duarte

_______________________________

Assinatura da pesquisadora responsável: Graziela De Luca Canto
